# A Rare Case of Foreign Body in Stomach: Dental Mouth Mirror

**Published:** 2018-02

**Authors:** Ramazan GÜVEN, Ufuk BARIS KUZU, Abdullah ŞENLIKÇI, Adnan BUDAK

**Affiliations:** 1. Dept. of Emergency, Bitlis State Hospital, Bitlis, Turkey; 2. Dept. of Gastroenterology, Bitlis State Hospital, Bitlis, Turkey; 3. Dept. of Surgery, Bitlis State Hospital, Bitlis, Turkey; 4. Dept. of General Director, Bitlis State Hospital, Bitlis, Turkey

## Dear Editor-in-Chief

Foreign body ingestion is a common condition in childhood, but it can also be seen in adults. Depending on the shape and size of the foreign body, it may cause serious complications such as perforation and obstruction. For this reason, foreign body ingestion management is vital (1).

**Fig. 1:**
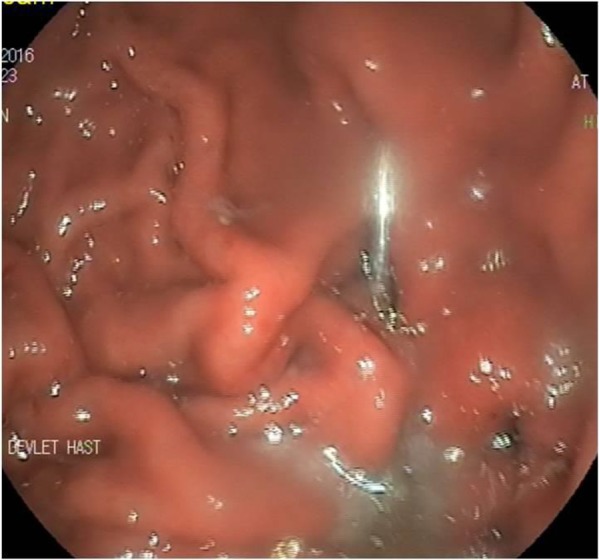
Mouth-mirror embedded in the corpus of the stomach

**Fig. 2:**
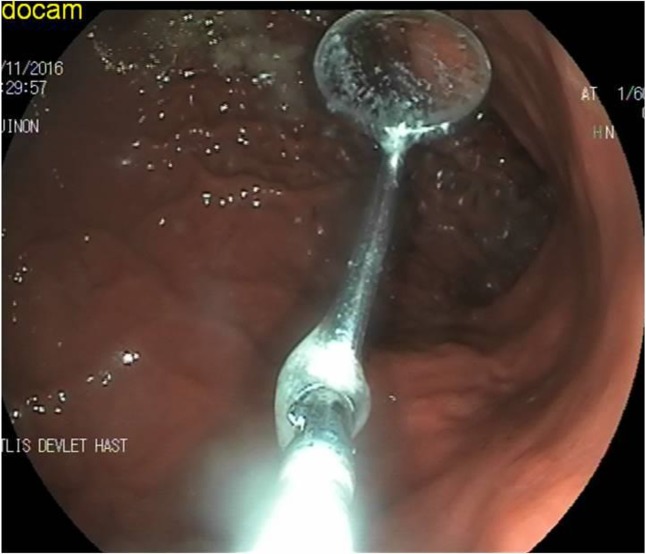
Mouth-mirror while it was pulled throughout the esophageal lumen

A 33-yr-old male patient refered to the Outpatient Clinic for a toothache. During the dental examination, the mouth mirror separated from the middle connection and divided into two, then the patient swallowed the head of the mirror. The patient had severe epigastric pain on emergency admission. An urgent endoscopic examination of the upper gastrointestinal tract was performed based on the patient's story. In the endoscopic examination, we found mouth mirror with a length of about 6 cm and its pointed part embedded in the corpus of the stomach (Fig. 1). The tip of the mirror was held with a forceps, and then it was pulled throughout the esophageal lumen and removed from the mouth (Fig. 2). In endoscopic examination, erosions were detected in the area of the gastric mucosa affected by the mirror. Free air was not detected in the standing abdominal X-ray, and then he was kept under observation for 24 h. The patient did not develop any complications while under observation was discharged later. Informed consent was taken from the patient.

One of the most common indications for emergency endoscopy is foreign body ingestion (2). In adults, foreign bodies ingestion is mostly related to the intake of solid food, but it may rarely occur during examination as in our case. For this reason, physicians and dentists should keep in mind that such complications may occur in the examination of the oral region. In addition, endoscopic procedures are effective methods for foreign bodies ingestion both in terms of diagnosis and treatment.
